# Supplementary figures and images for: Chemotherapy activates inflammasomes to cause inflammation-associated bone loss
Source: eLife. 2024 Apr 11;13:RP92885. doi: 10.7554/eLife.92885 (PMC11008812; doi:10.7554/eLife.92885)

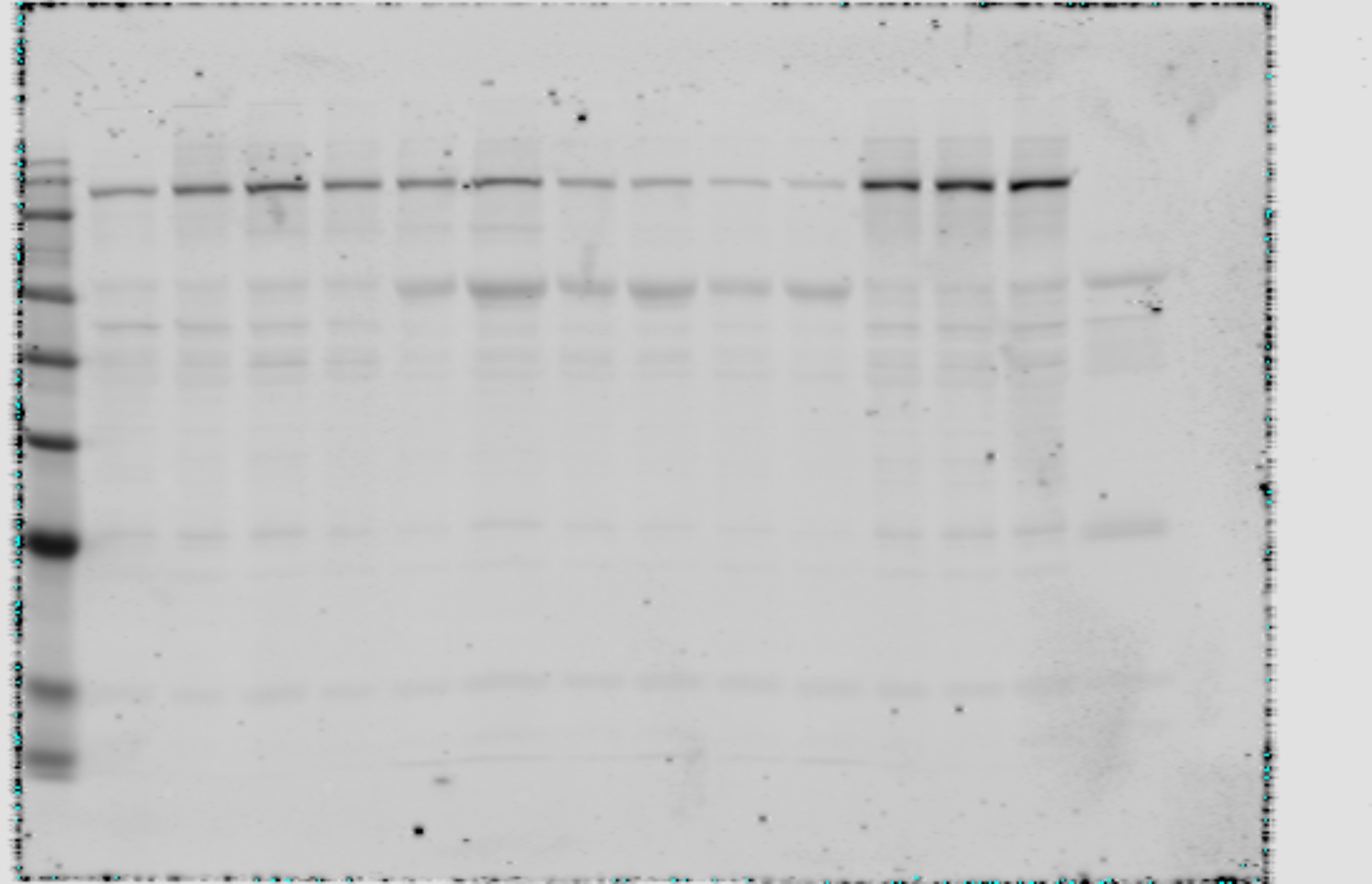

Supplement: Figure 4—source data 1. [file elife-92885-fig4-data1.zip › Figure 4-source data 1.tif]

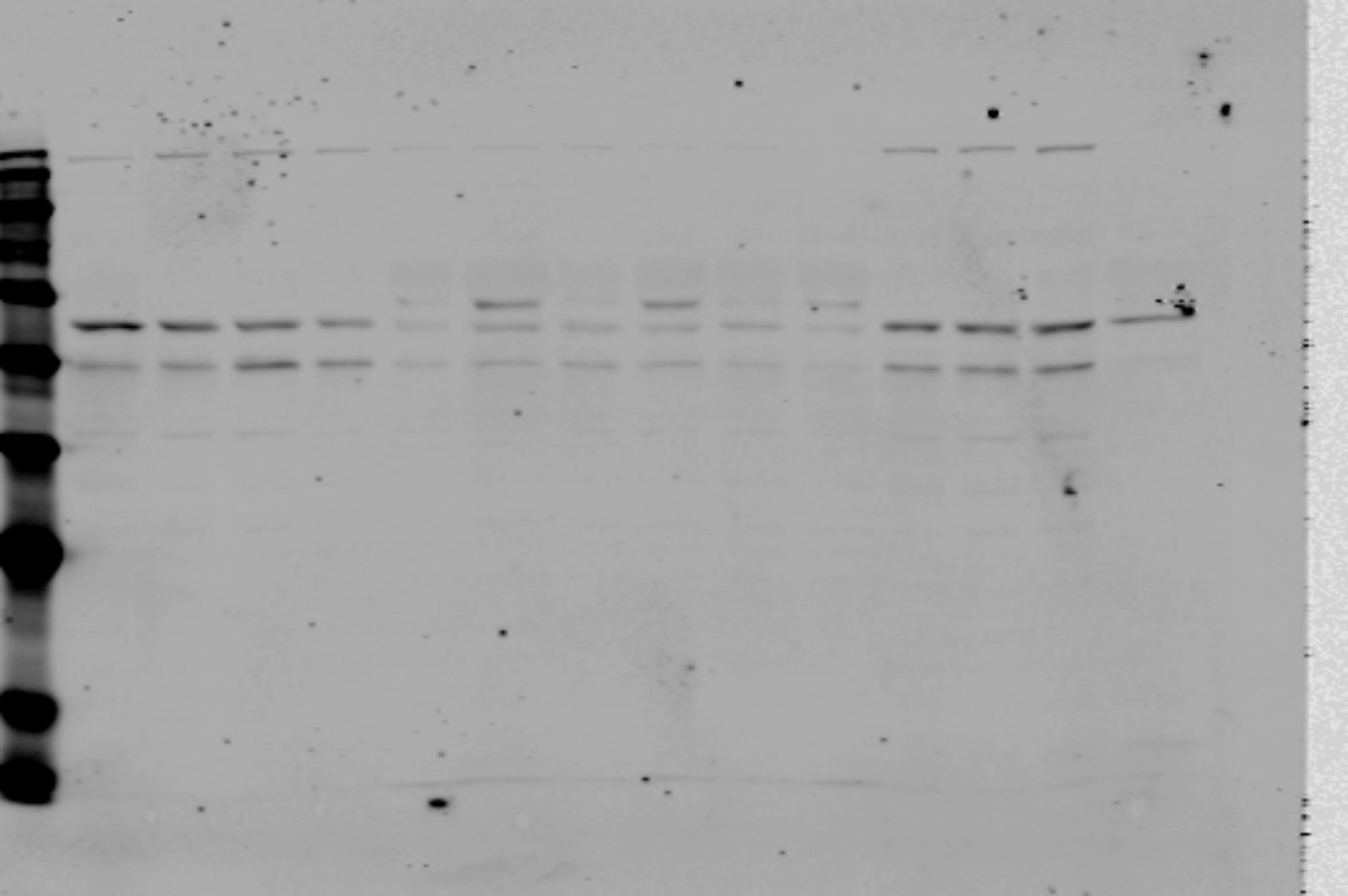

Supplement: Figure 4—source data 2. [file elife-92885-fig4-data2.zip › Figure 4-source data 2.tif]

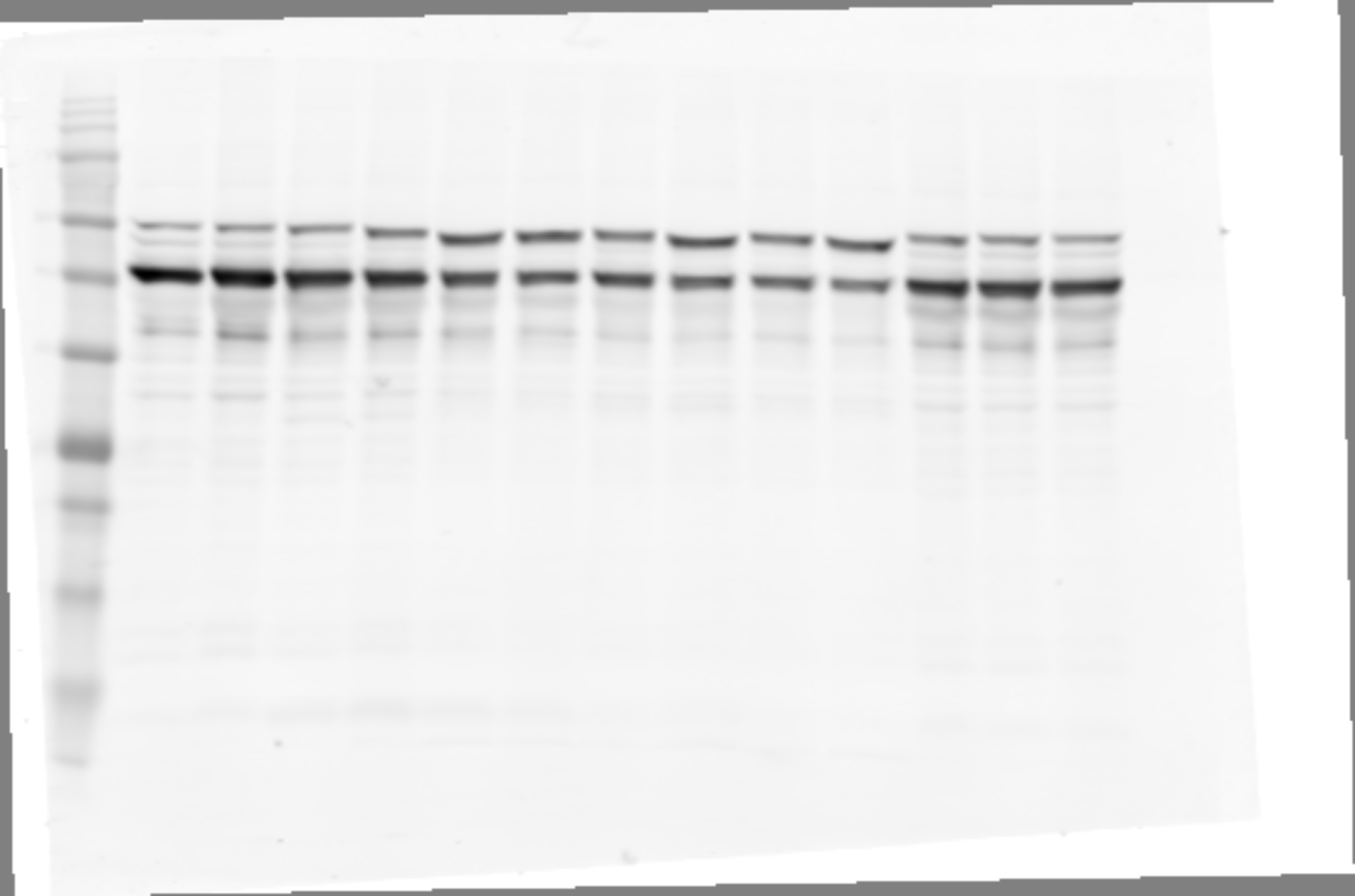

Supplement: Figure 4—source data 3. [file elife-92885-fig4-data3.zip › Figure 4-source data 3.tif]

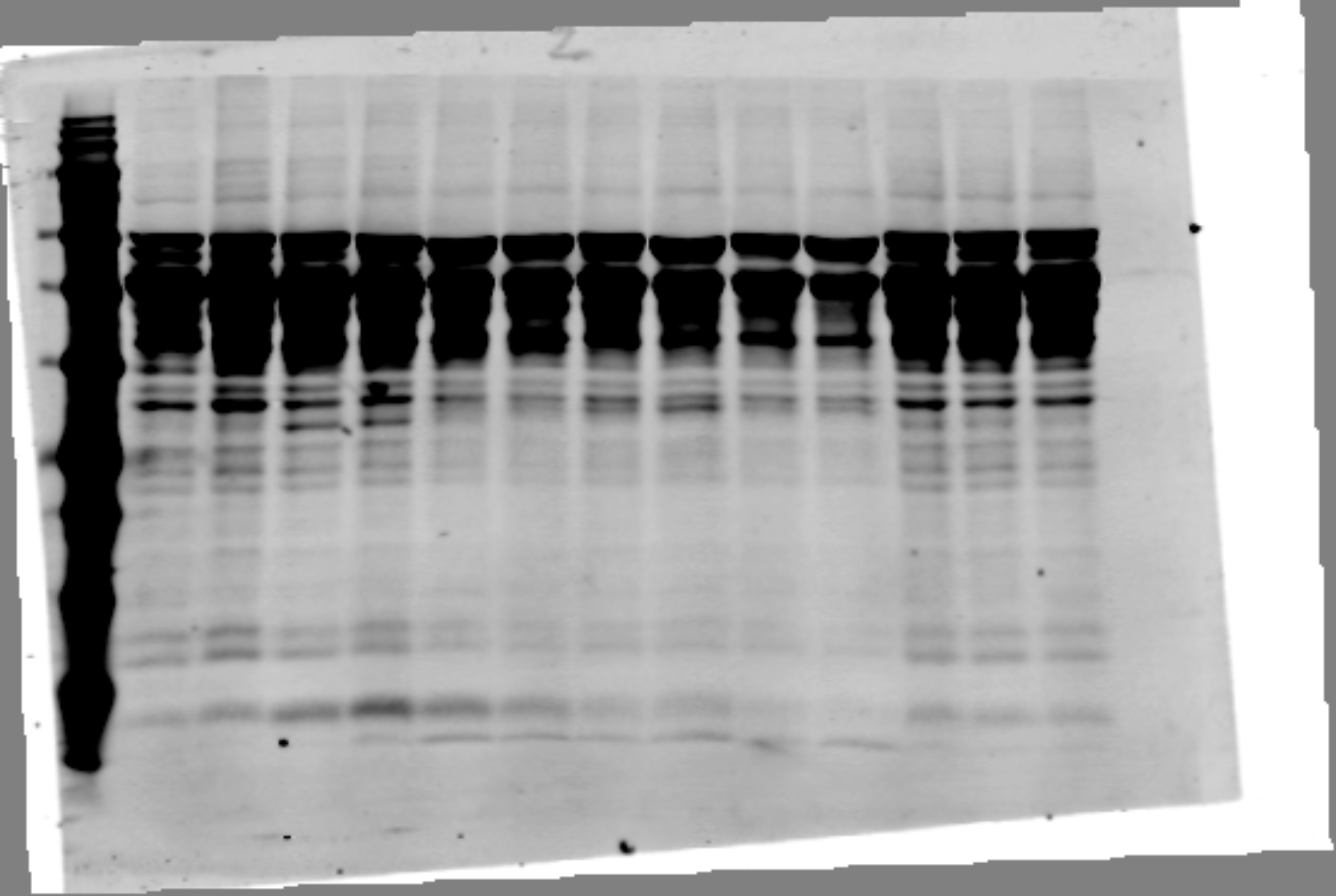

Supplement: Figure 4—source data 4. [file elife-92885-fig4-data4.zip › Figure 4-source data 4.tif]

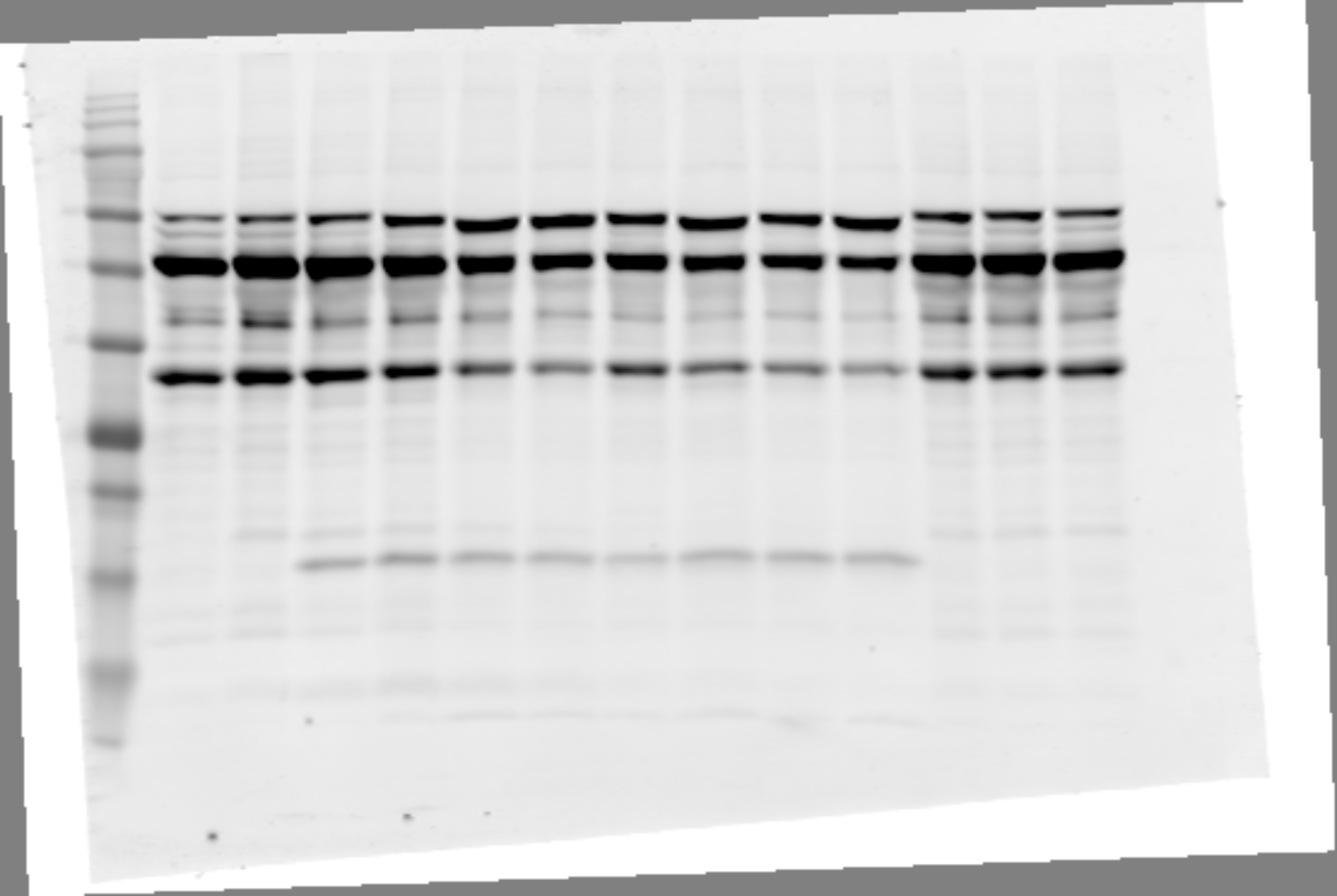

Supplement: Figure 4—source data 5. [file elife-92885-fig4-data5.zip › Figure 4-source data 5.tif]

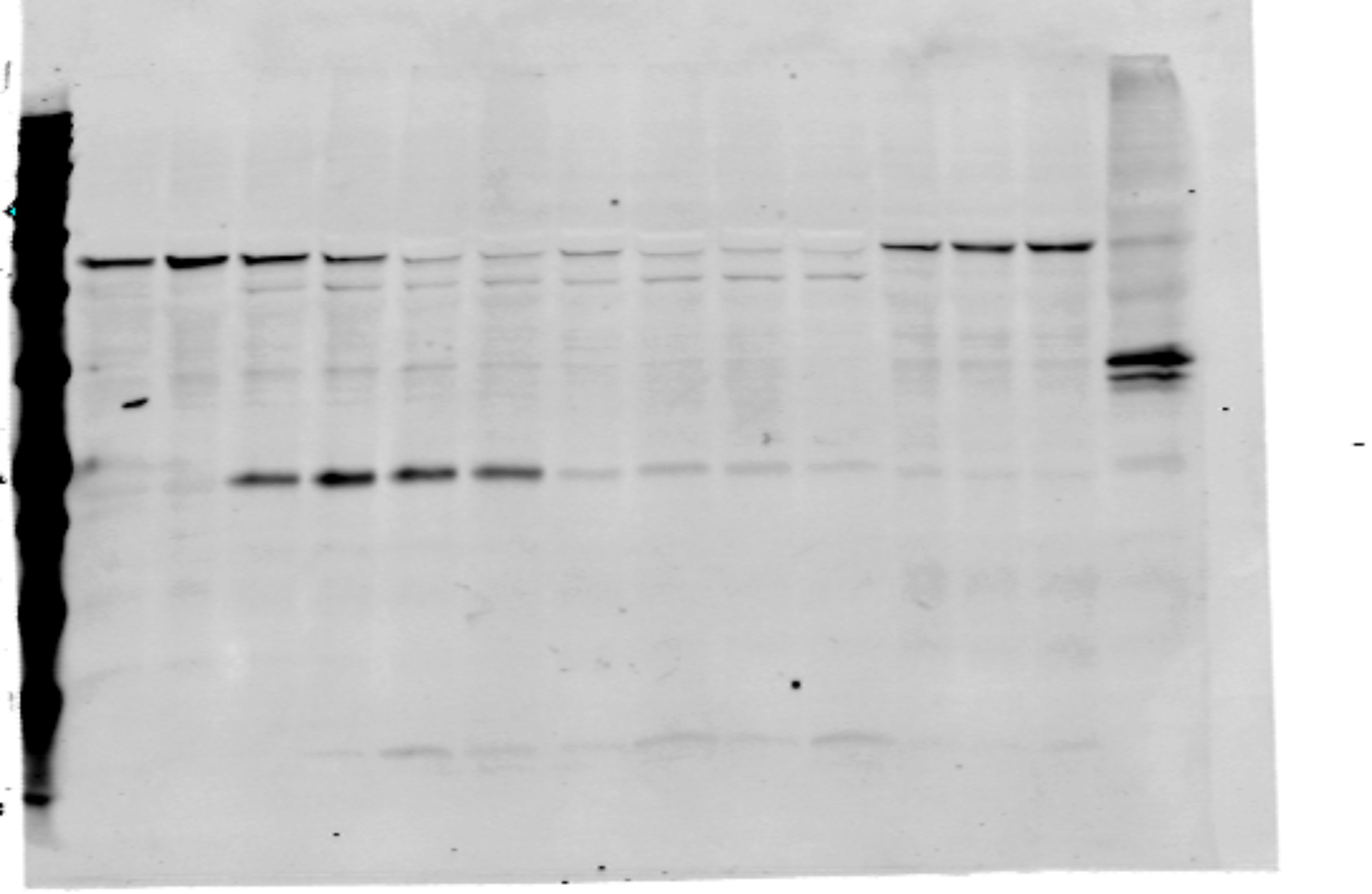

Supplement: Figure 4—source data 6. [file elife-92885-fig4-data6.zip › Figure 4-source data 6.tif]

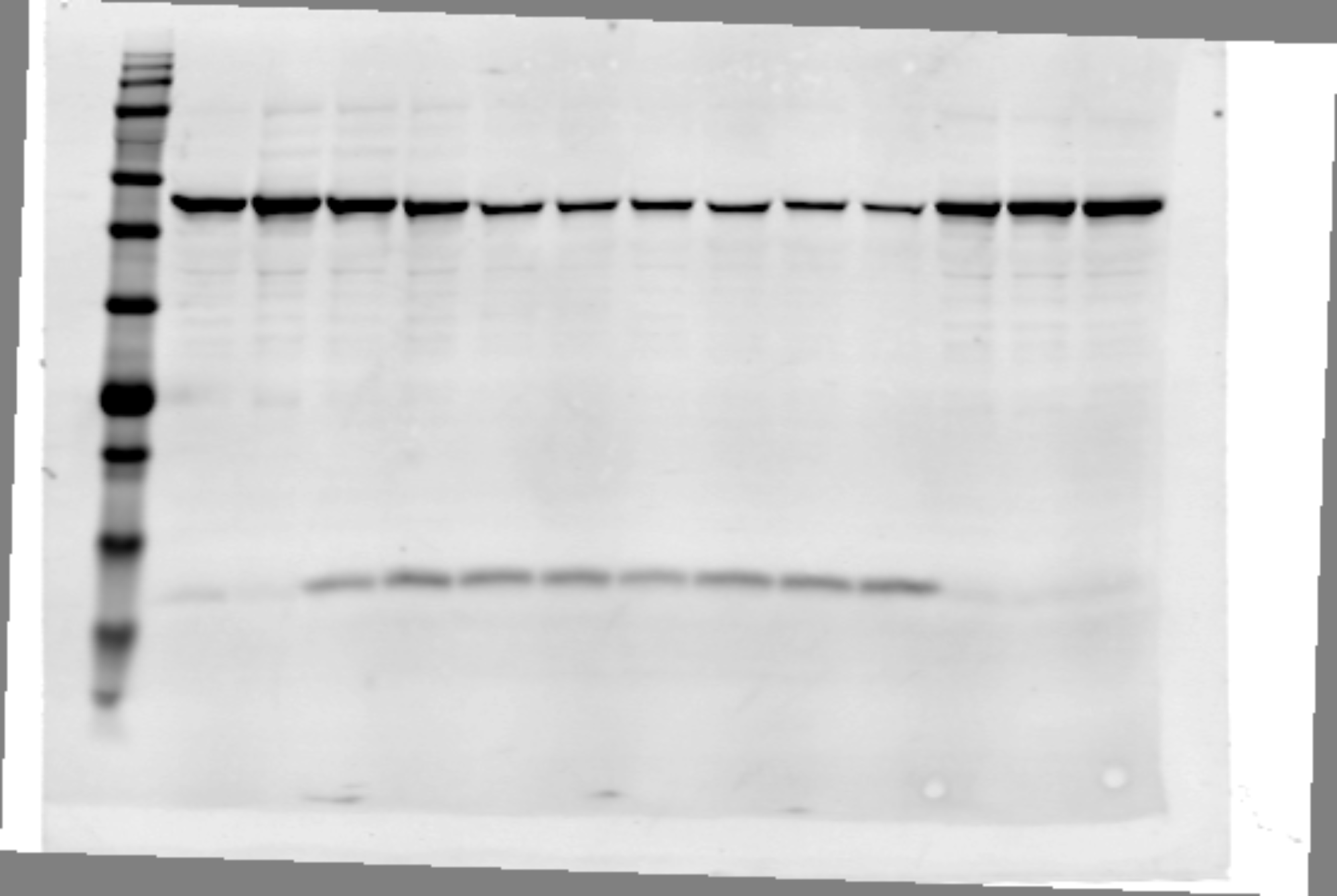

Supplement: Figure 4—source data 7. [file elife-92885-fig4-data7.zip › Figure 4-source data 7.tif]

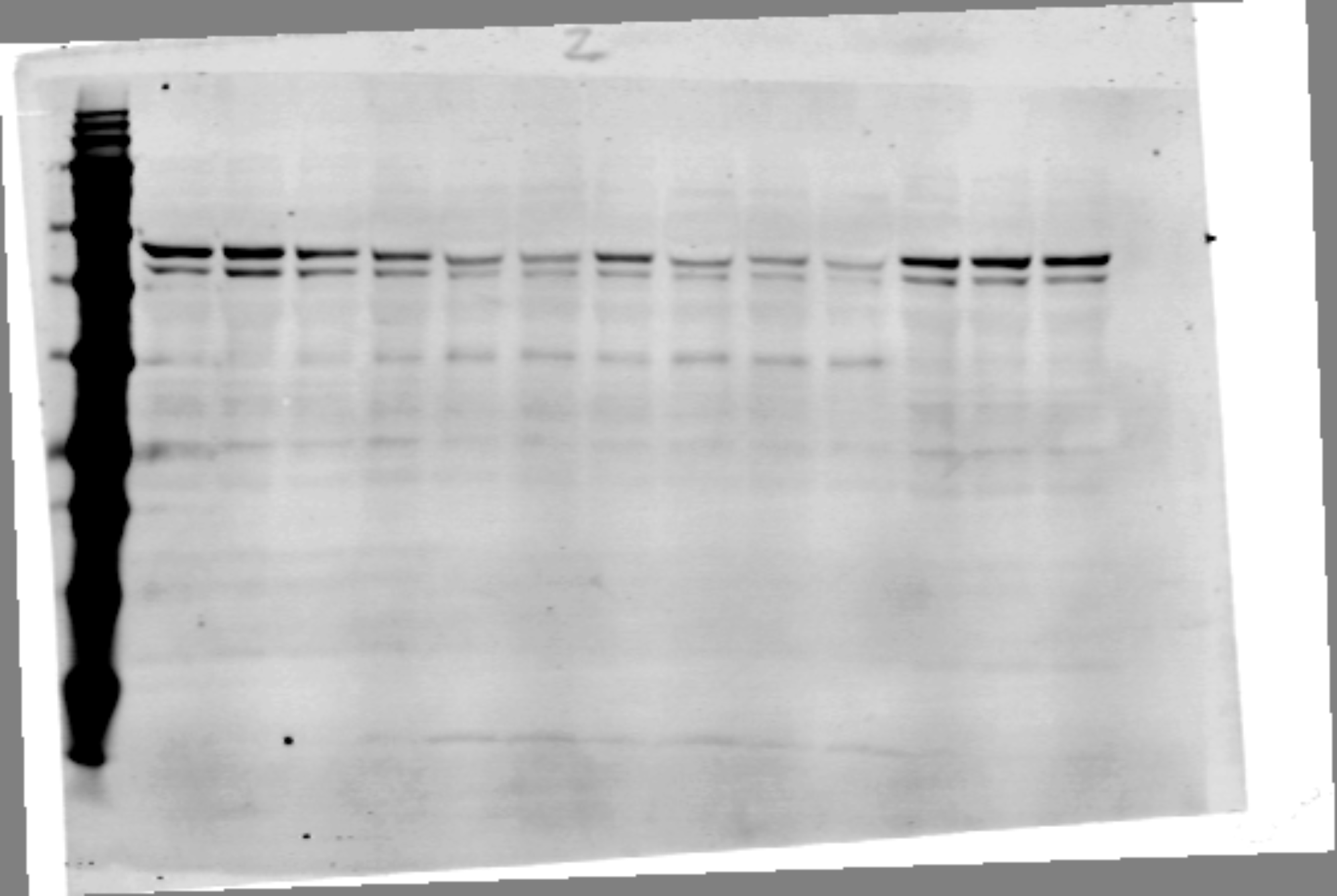

Supplement: Figure 4—source data 8. [file elife-92885-fig4-data8.zip › Figure 4-source data 8.tif]

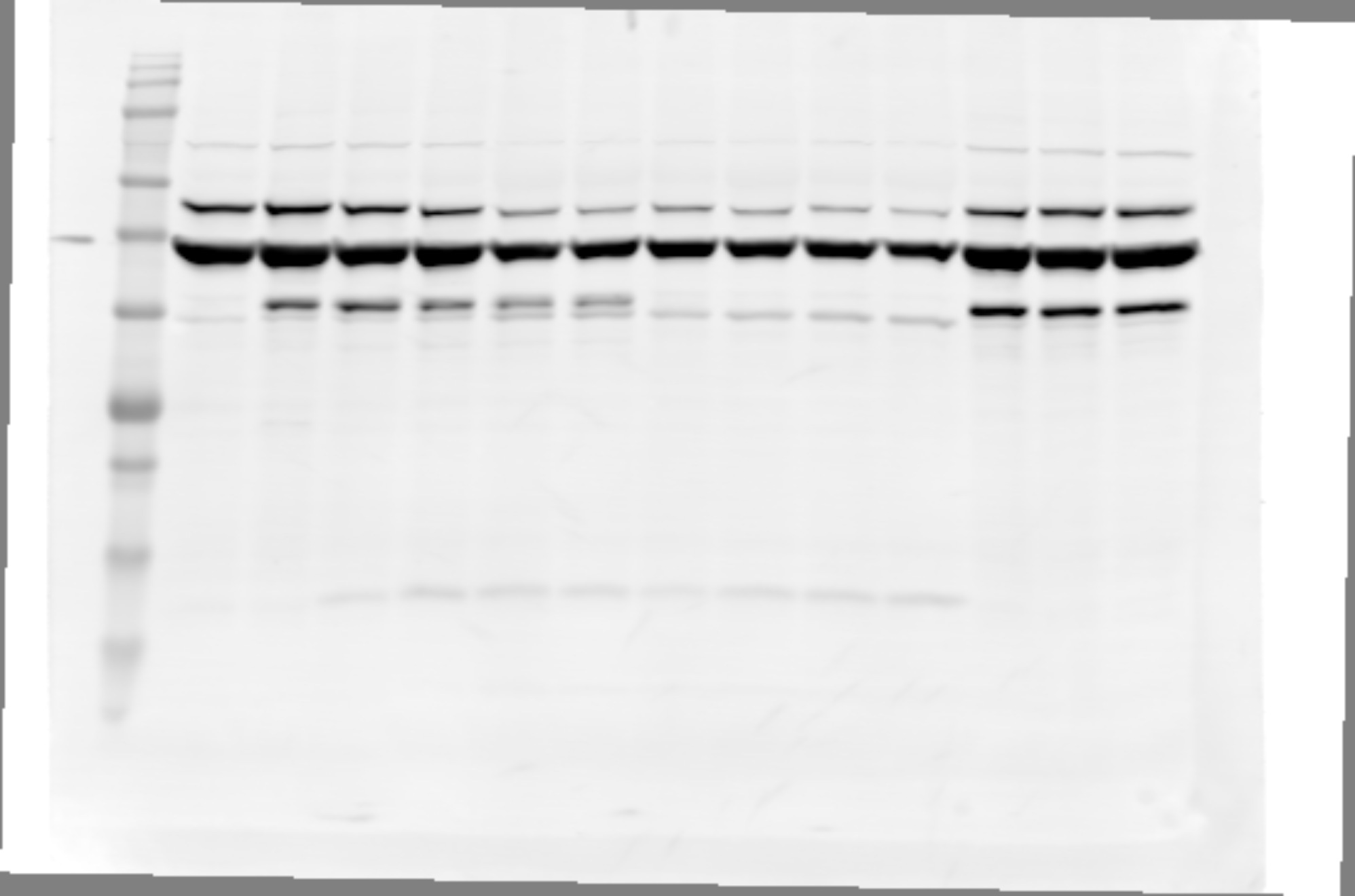

Supplement: Figure 4—source data 9. [file elife-92885-fig4-data9.zip › Figure 4-source data 9.tif]

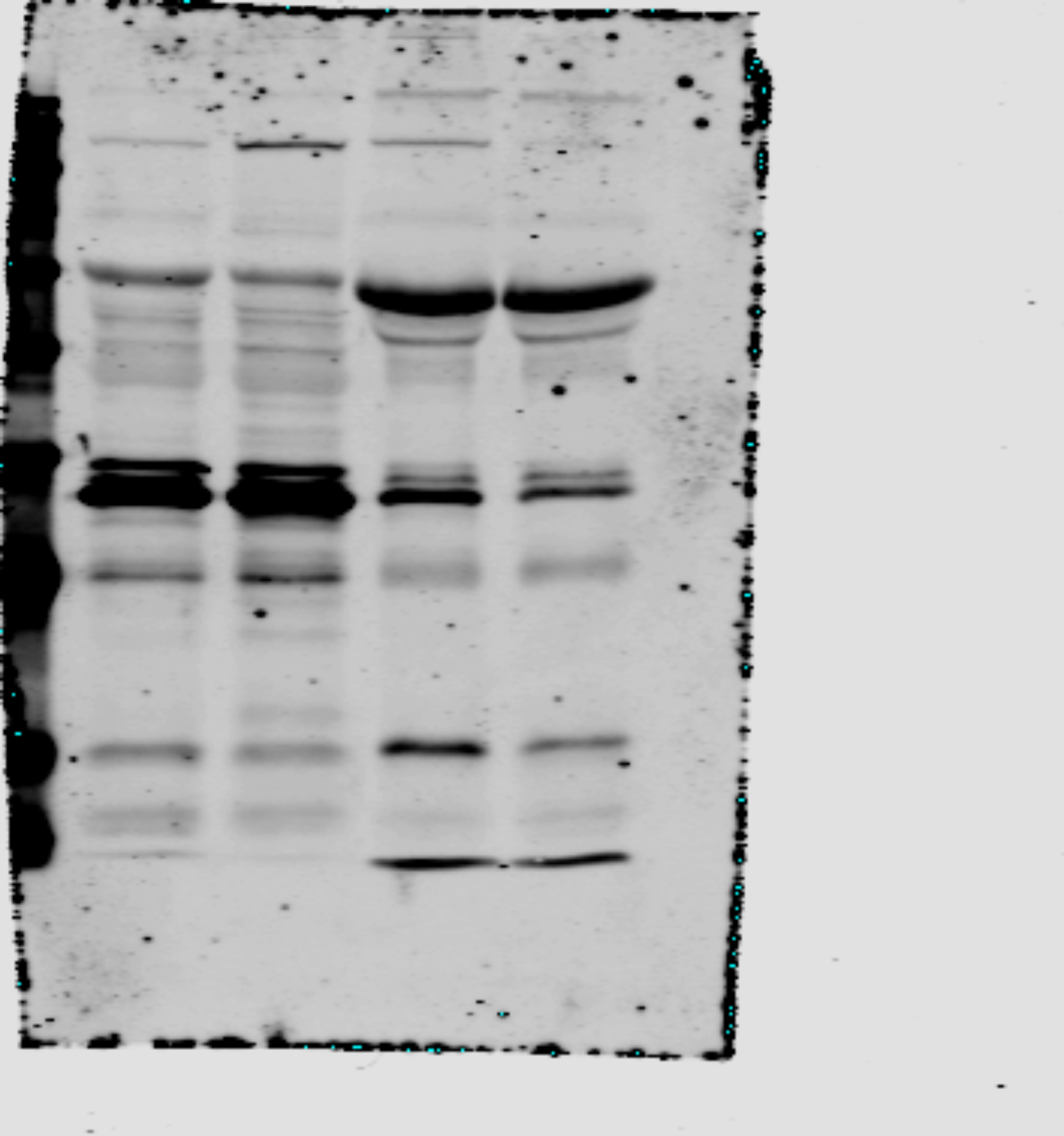

Supplement: Figure 5—source data 1. [file elife-92885-fig5-data1.zip › Figure 5-source data 1.tif]

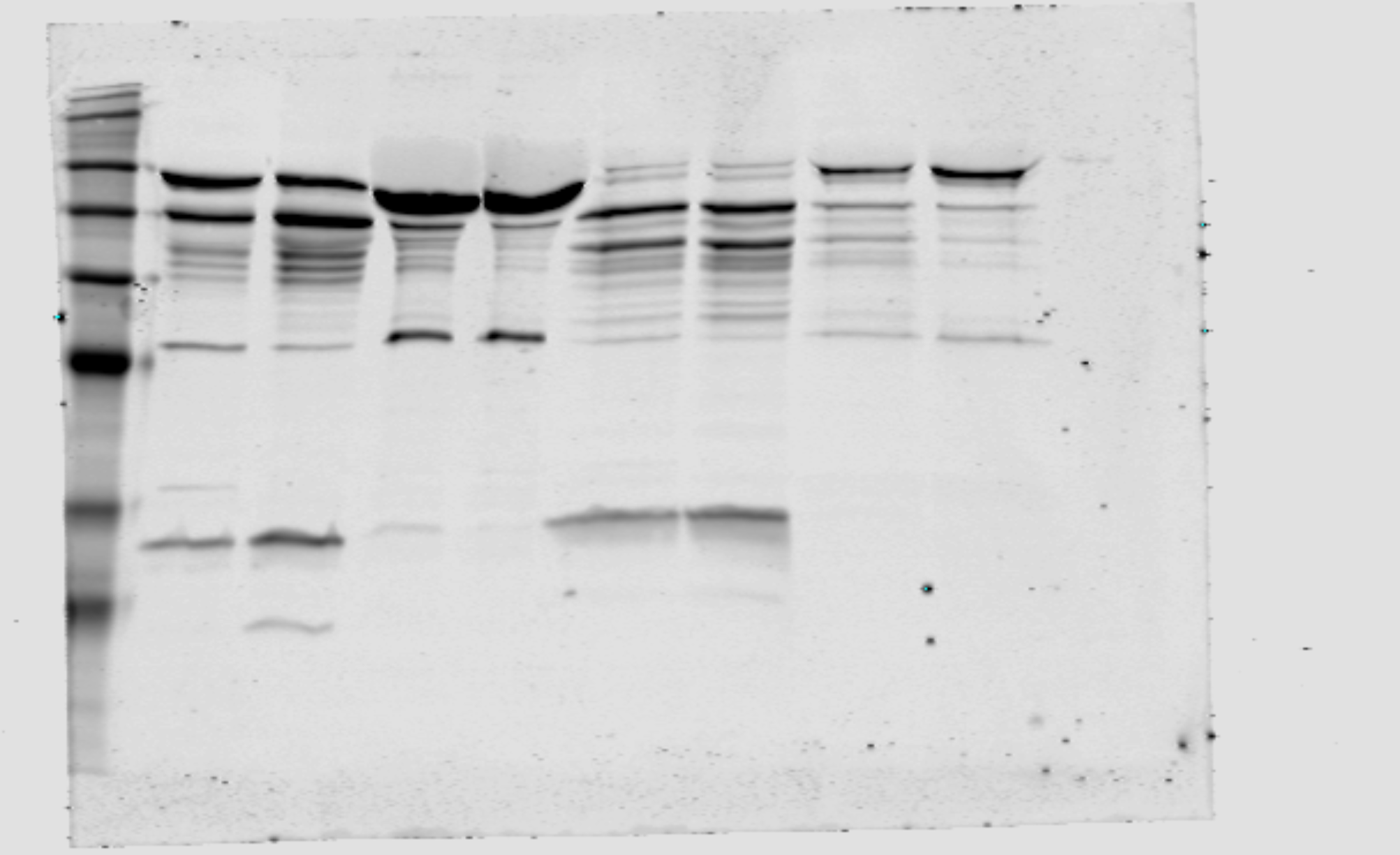

Supplement: Figure 5—source data 2. [file elife-92885-fig5-data2.zip › Figure 5-source data 2.tif]

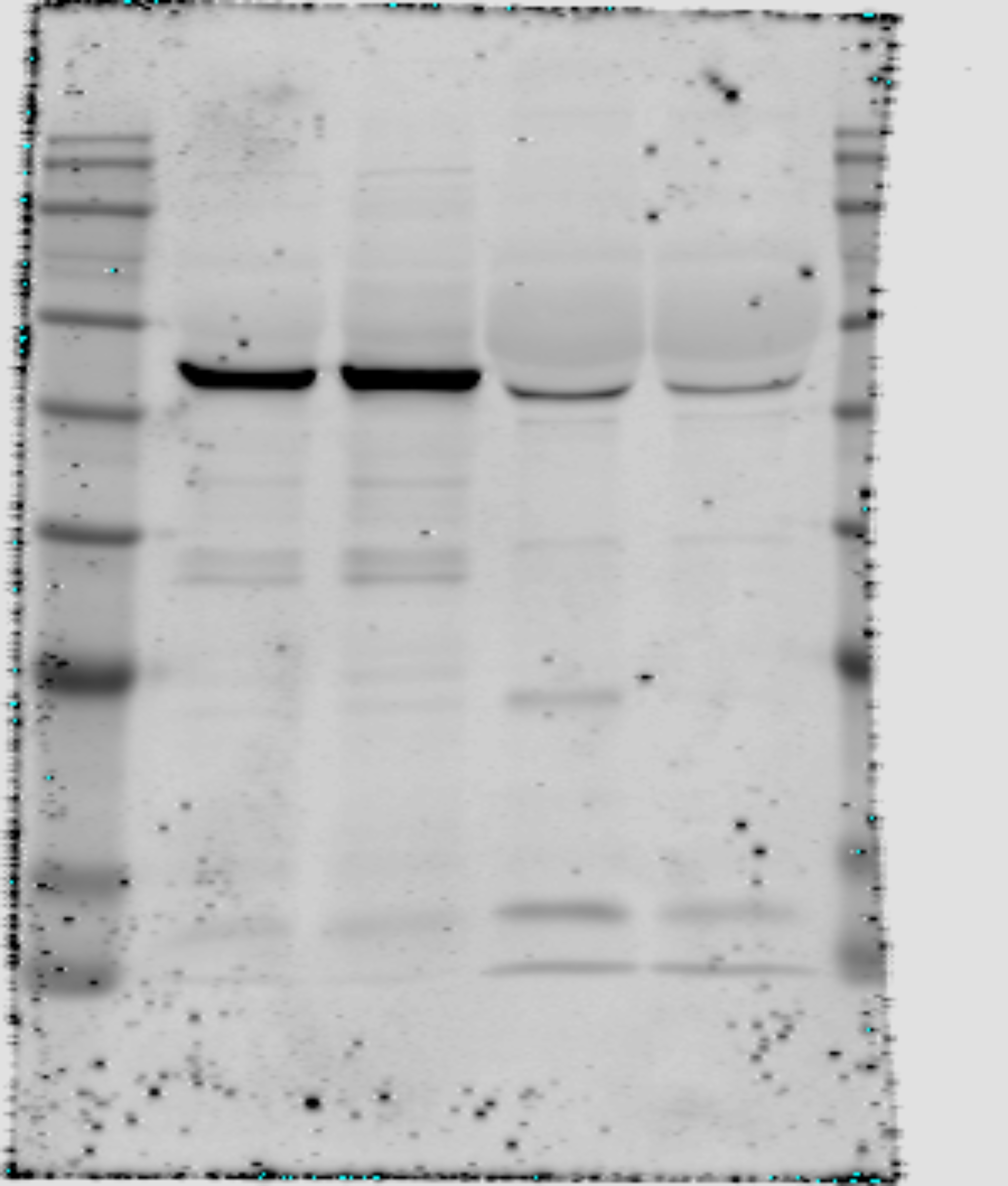

Supplement: Figure 5—source data 3. [file elife-92885-fig5-data3.zip › Figure 5-source data 3.tif]

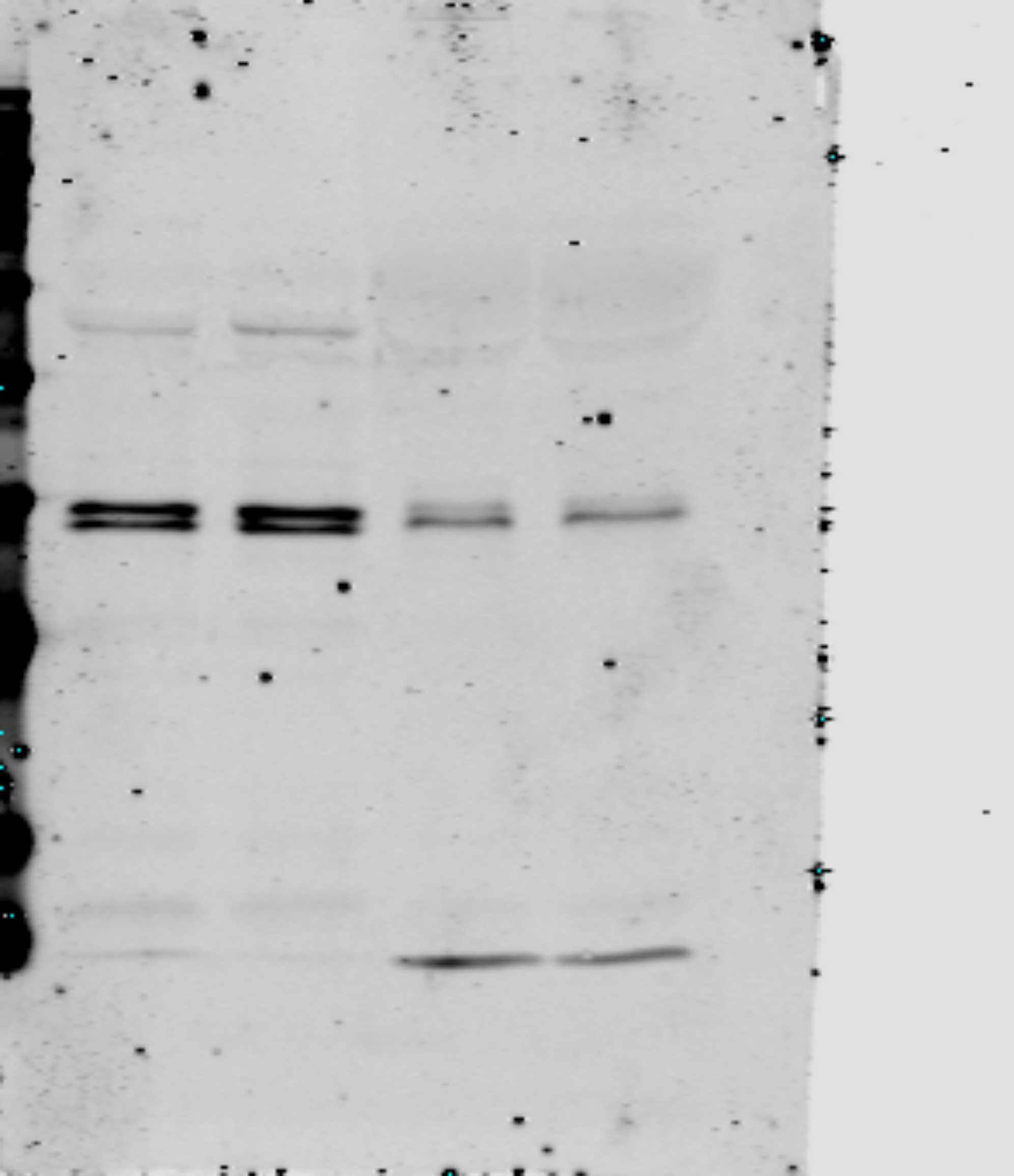

Supplement: Figure 5—source data 4. [file elife-92885-fig5-data4.zip › Figure 5-source data 4.tif]

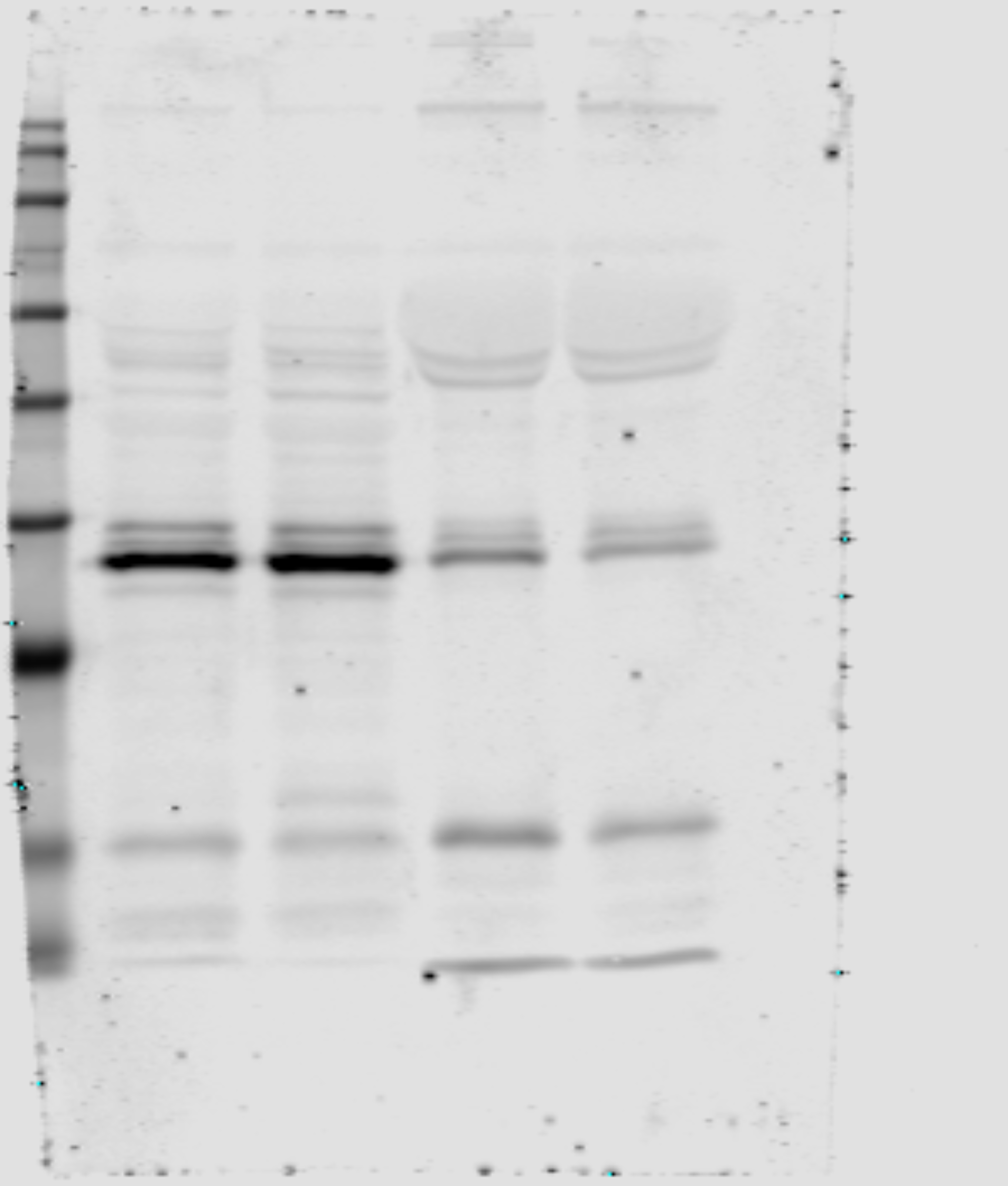

Supplement: Figure 5—source data 5. [file elife-92885-fig5-data5.zip › Figure 5-source data 5.tif]

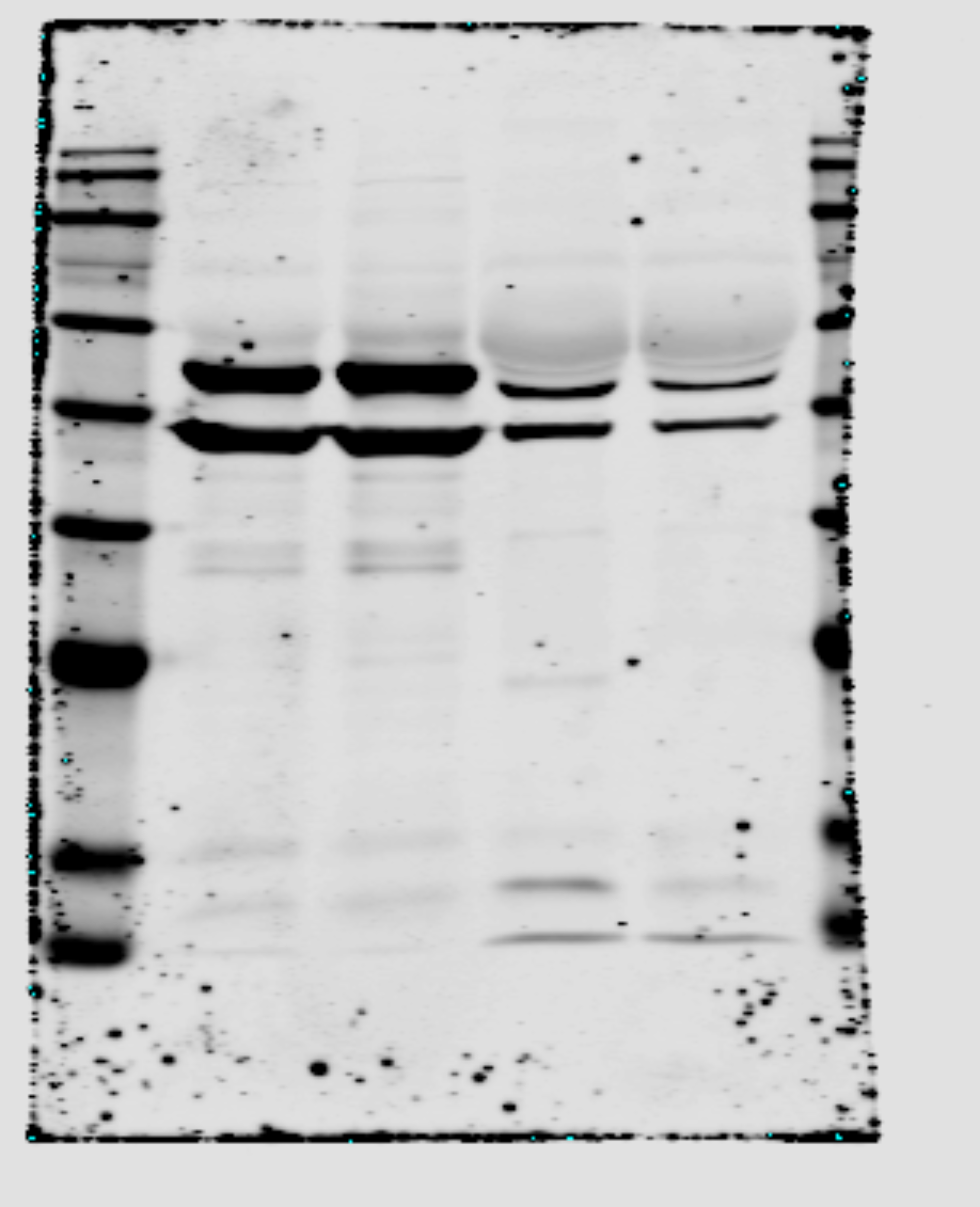

Supplement: Figure 5—source data 6. [file elife-92885-fig5-data6.zip › Figure 5-source data 6.tif]

# 1. NLRP3

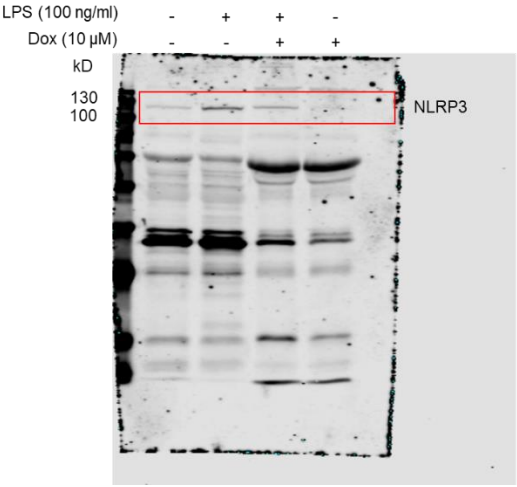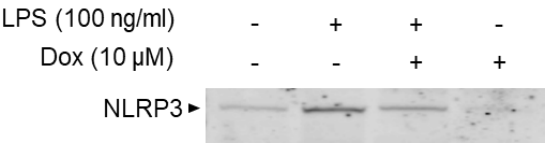

# 2. Caspase-1

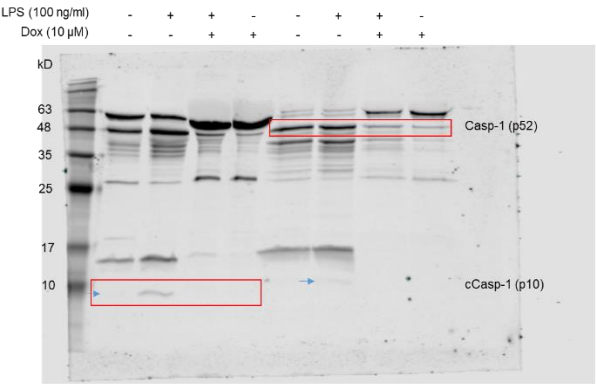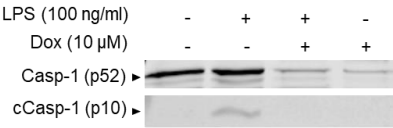

# 3. Gasdermin D (GSDMD)

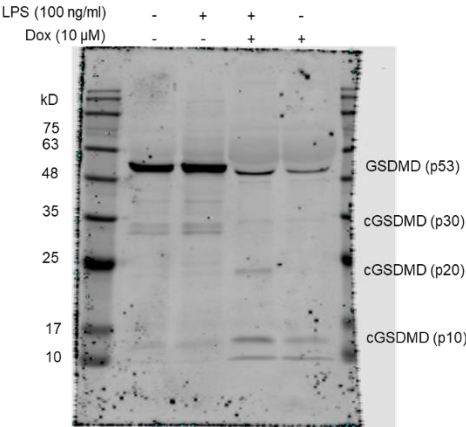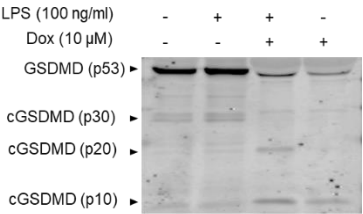

# 4. Gasdermin E (GSDME)

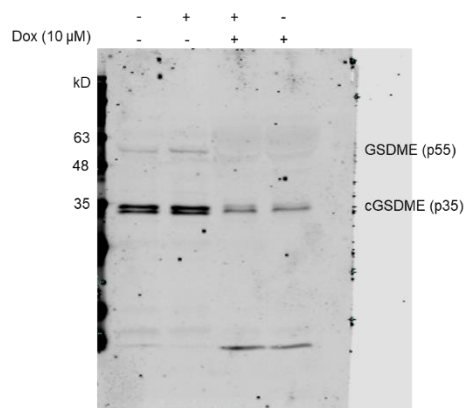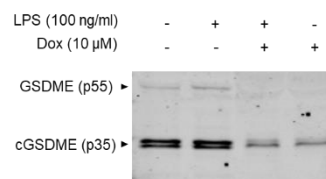

## 5. Caspas-3

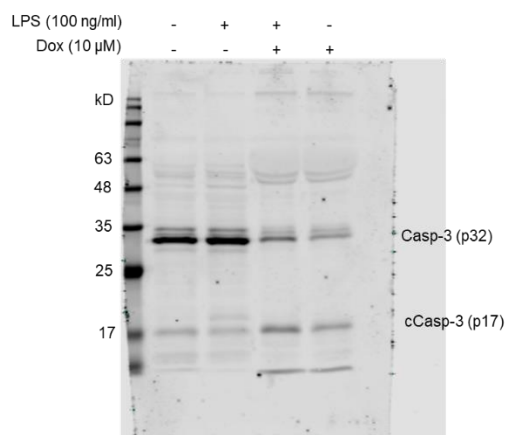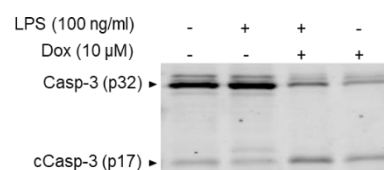

## 6. $\beta$ -actin

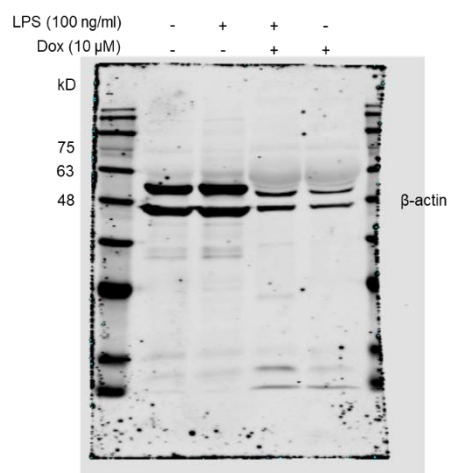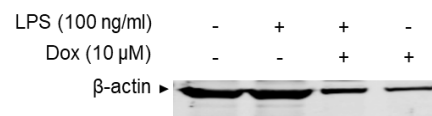

Supplement: Figure 5—source data 7. [file elife-92885-fig5-data7.zip › Figure 5- source data 7.pdf]
