# Supplementary material for: Chemotherapy activates inflammasomes to cause inflammation-associated bone loss
Source: eLife. 2024 Apr 11;13:RP92885. doi: 10.7554/eLife.92885 (PMC11008812; doi:10.7554/eLife.92885)

1. **NLRP3**

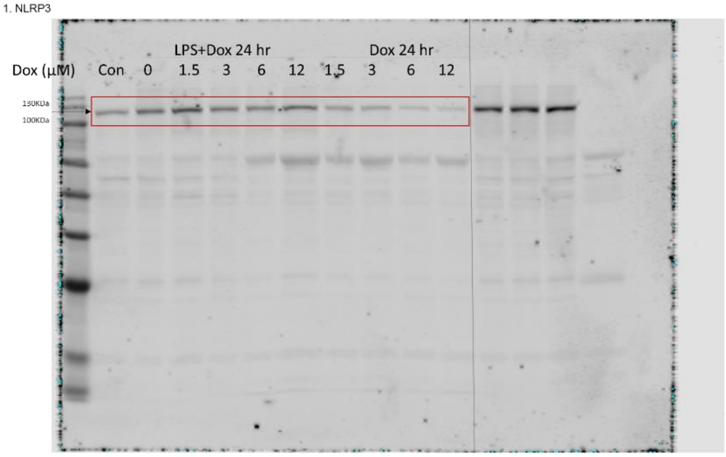

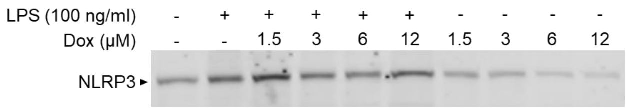

2. **AIM2**

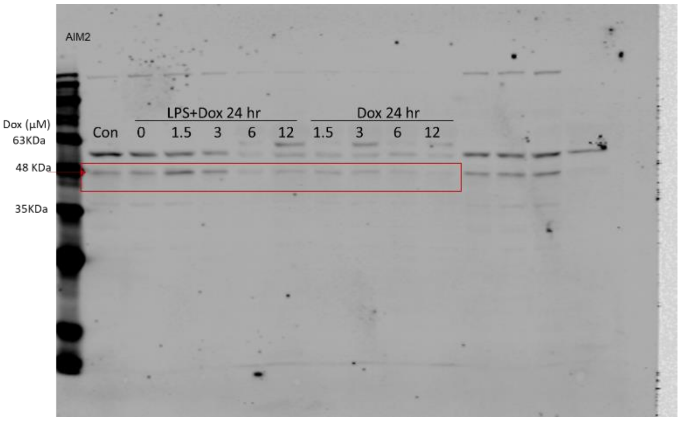

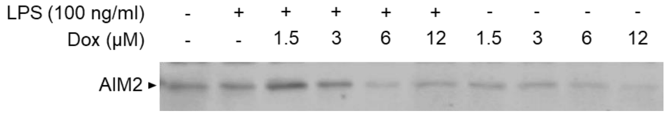

3. **Caspase-1**

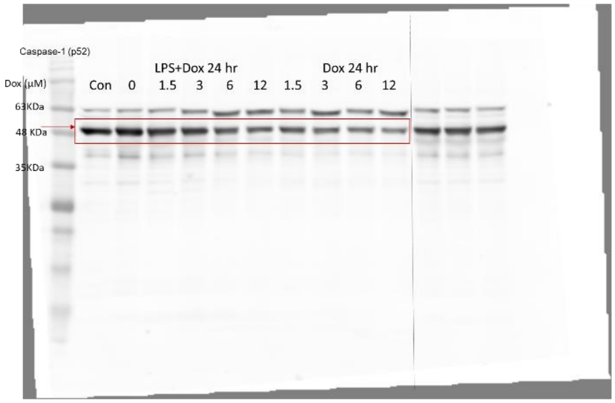

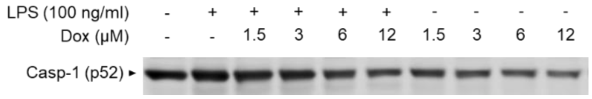

4. **Cleaved-caspase-1 (high intensity of caspase-1 blot)**

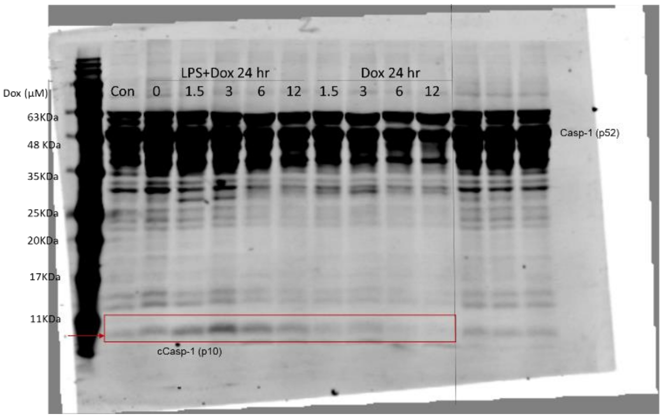

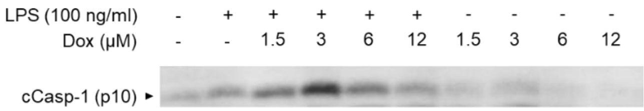

5. Caspase-3

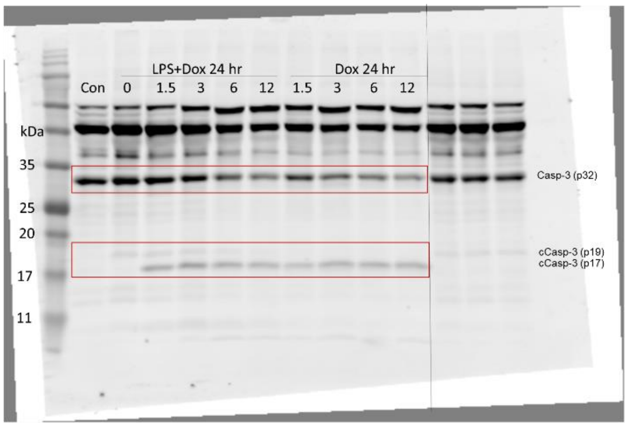

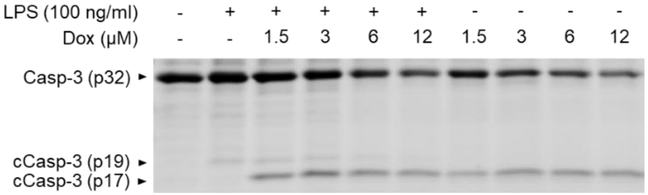

6. Gasdermin D (GSDMD)

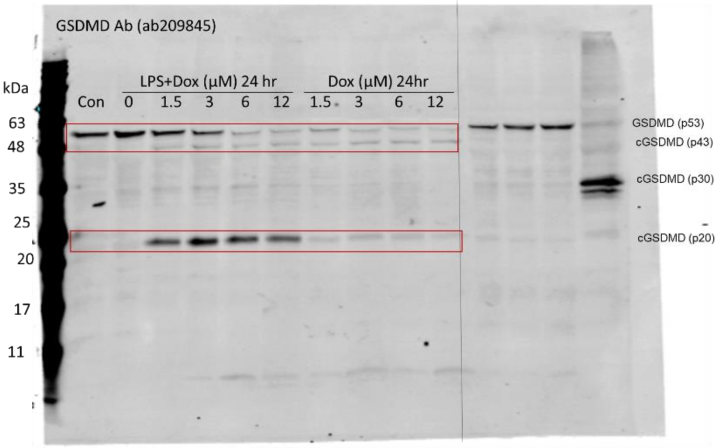

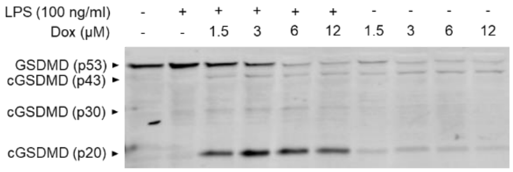

7. Gasdermin D (GSDMD)

GSDMD Ab: ab219800

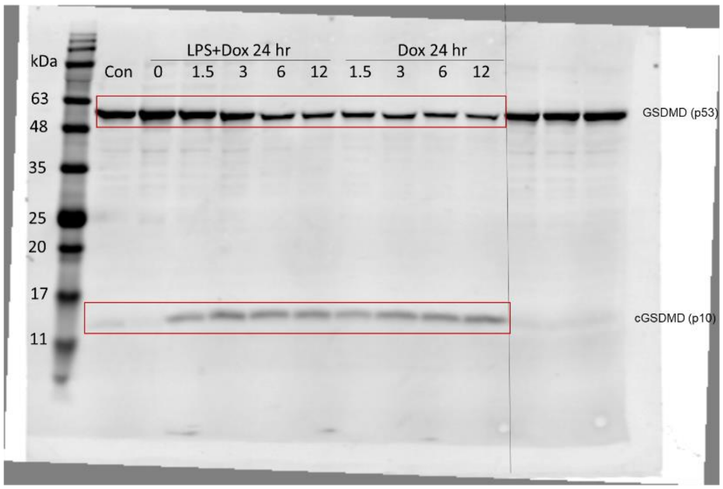

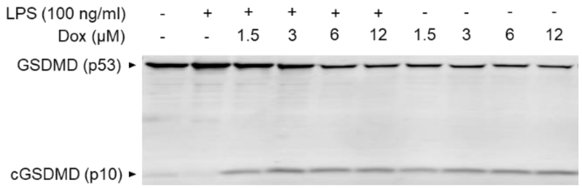

8. Gasdermin E (GSDME)

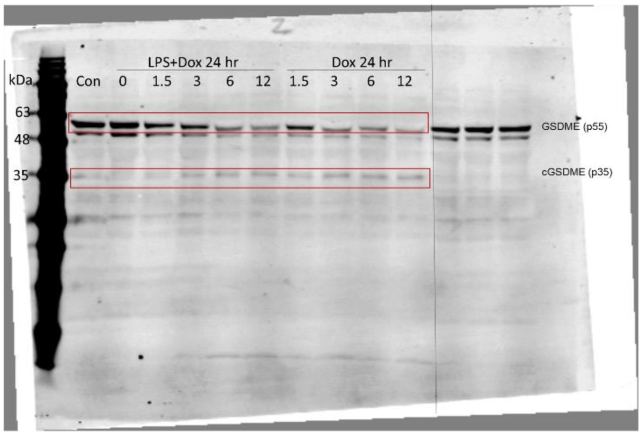

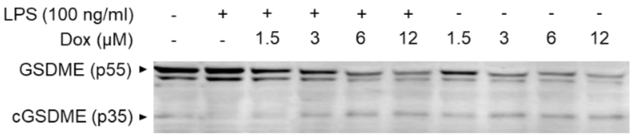

9. β-actin

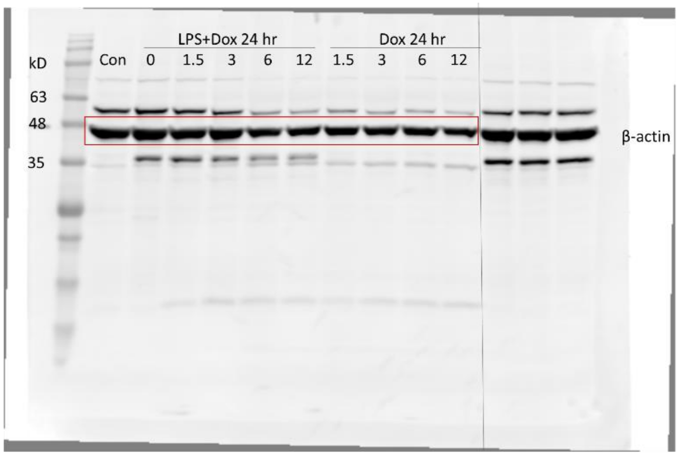

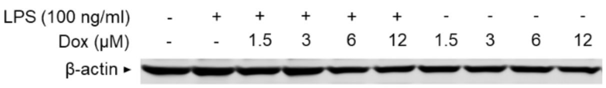

Supplement: Figure 4—source data 10. [file elife-92885-fig4-data10.zip › Figure 4- source data 10.pdf]
